# Interobserver and Intraobserver Reliabilities of Three-Dimensional Postoperative Evaluation Software in Total Hip Arthroplasty

**DOI:** 10.1155/2021/3705789

**Published:** 2021-09-16

**Authors:** Kenta Konno, Shigeo Hagiwara, Junichi Nakamura, Yuya Kawarai, Sumihisa Orita, Yawara Eguchi, Seiji Ohtori

**Affiliations:** Department of Orthopaedics Surgery, Graduate School of Medicine, Chiba University, 1-8-1 Inohana, Chuo-ku, Chiba 260-8670, Japan

## Abstract

**Background:**

In primary and revision total hip arthroplasty, to prevent perioperative complications, the prediction of the optimal implant size is essential. Using three-dimensional computed tomography-based postoperative evaluation software, we explored the accuracy and the precision of measurement of the size, alignment, and position of the prosthetic components after total hip arthroplasty.

**Methods:**

Using postoperative evaluation software, the postoperative computed tomography data from 20 hips in 20 patients were evaluated. The component size, alignment, and three-dimensional positioning of the cup and stem were assessed. The concordance rates of the component, repeatability (intraobserver reliability), and reproducibility (interobserver reliability) of postoperative evaluation were calculated. The radiographic inclination and radiographic anteversion of the cup, anteversion, varus–valgus angle, and flexion–extension angles of the stem were measured for alignment. The implant positioning was measured along three axes, namely, *X*-axis (transverse), *Y*-axis (sagittal), and *Z*-axis (longitudinal).

**Results:**

The concordance rates of all parts are above 94%. The intraobserver and interobserver intraclass correlation coefficients of alignment measurement were very good for both cup (0.879–0.964) and stem (0.973–0.996). The intraobserver and interobserver intraclass correlation coefficients of cup positioning were very good (0.961–0.987) for all axes. The intraobserver and interobserver intraclass correlation coefficients of implant positioning were very good for the stem (0.879–0.995) for all axes.

**Conclusions:**

Computed tomography-based postoperative evaluation software was able to evaluate the size and position of total hip implants with high reproducibility.

## 1. Introduction 

In primary and revision total hip arthroplasty (THA), appropriate implant size and placement are necessary to prevent complications such as dislocation [1, 2], wear of the polyethylene liner, and loosening of the components [3–5]. Lewinnek et al. proposed a “safe zone” of cup alignment, and Widmer et al. proposed an optimal combination of cup alignment and stem anteversion to minimize the risk of impingement [6]. To achieve precise implant placement, the use of a navigation system and a fluoroscopy-guided technique have been reported [7, 8]. Present, the gold standard for the postoperative assessment of component orientation, inclination, anteversion of the acetabular component, and varus–valgus angle of the femoral component is conventional plain radiography [9–11]. Although using these measurement methods, implant alignment accurately can be evaluated, these methods can be less reliable in assessing the components' exact size, position, and alignment in the acetabulum and femur [12, 13] because evaluating three-dimensional (3D) implant alignment using a two-dimensional plain radiograph or intraoperative measurement is challenging [1, 3]. In the case of revision THA without detail implant size and medical record due to long periods of time or moving, predicting the implant size and alignment accurately would be very helpful. It has been reported that a computed tomography- (CT-) based method using low-dose digital stereoradiography, commercialized as the EOS imaging system, has good reliability [14–16].

Recently, a CT-based 3D preoperative planning and postoperative evaluation software, which can measure the alignment and 3D position of the implant, has become available and has shown good reliability for preoperative planning [17, 18]. In total knee arthroplasty, good reliability has been reported regarding the femoral and tibial component positions and alignment [19]. Although the reliability of measurement might be a part of the clinical study, no study has reported the whole reliability of the evaluation software, including component size, alignment, and three-dimensional positioning. The present study aimed to evaluate the accuracy, intraobserver reliability, and interobserver reliability of the CT-based postoperative evaluation software in THA.

## 2. Materials and Methods

### 2.1. Study Design

We conducted a retrospective study using CT-based 3D preoperative planning and postoperative analysis software for THA. The study protocol was in compliance with the Helsinki Declaration, approved by the institutional review board, and all patients provided written informed consent before any study-related procedures were conducted.

### 2.2. Subjects

We selected perioperative CT data from 20 hips in 20 patients who underwent primary THA for osteoarthritis (OA) of the hip and osteonecrosis of the femoral head (ONFH). The planning and operations were all performed by one senior surgeon (N.J.). All patients underwent a unilateral THA between March 2016 and December 2017. Fifteen right and five left hips in eight male and 12 female patients were included in the present study. R3 cementless acetabular cup (Smith and Nephew, Memphis, TN) and Profemur *Z* cementless stem (Microport Orthopedics, Arlington, TN) were implanted in all cases. Exclusion criteria for this study included previous hip surgery including THA, osteotomy, and osteosynthesis, subluxation of Crowe type 2 or greater, and ankylosis. There were 15 hips with OA and five hips with ONFH each in stage 3B and stage 4 of the Japanese Ministry of Health, Labor, and Welfare stage classification [20].  Table 1 provides the mean age at postoperative CT scan, mean height, body weight, and body mass index. The shape of the femoral canal [21] was classified as champagne flute (canal flare index (CFI) > 4.7) in three hips, normal (3.0 ≤ CFI ≤ 4.7) in 14 hips, and stovepipe (CFI < 3.0) in three hips.

### 2.3. Planning and Analysis

**Figure 1:**
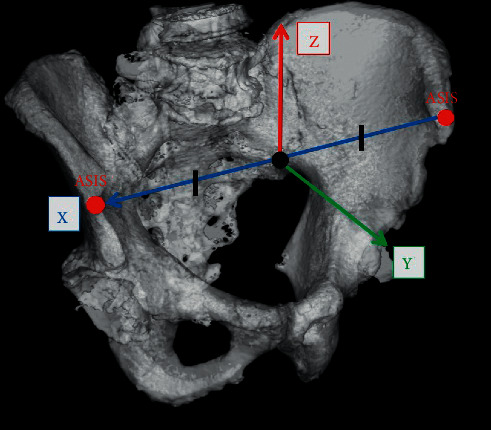
Functional pelvic plane coordinate system. The origin (white point) coincident with the middle point of the left and right anterior superior iliac spine (ASIS).

**Figure 2:**
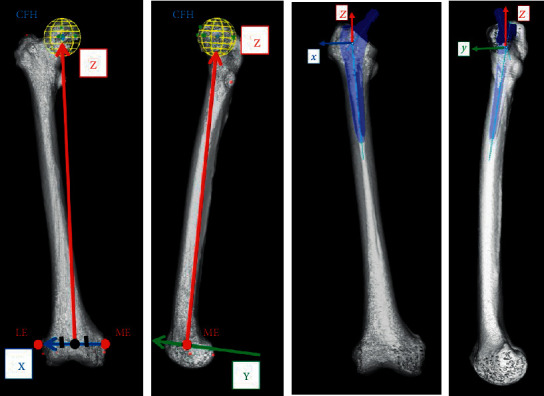
The femoral coordinate system recommended by the International Society of Biomechanics (ISB). The origin (black point) coincident with the middle point of the medial femoral epicondyle (ME) and lateral femoral epicondyle (LE).

**Figure 3:**
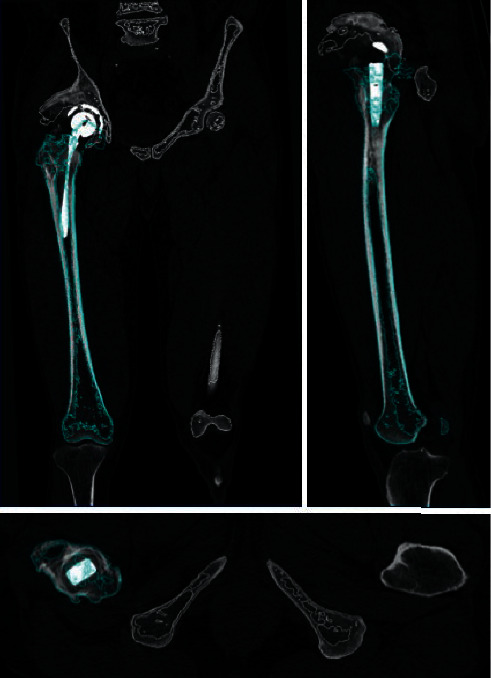
Image matching of the preoperative and postoperative CT images. Preoperative CT images (blue line) were fused to the postoperative images (white line) automatically as bone surfaces matched.

Both preoperative and postoperative CT scans from the bilateral iliac wing to the tibial plateau were performed with a slice thickness of 1 mm using a helical CT scanner (Aquilion ONE; TOSHIBA Medical Systems Corporation, Tokyo, Japan). The CT data were transferred to ZedHip (Lexi Corporation, Tokyo, Japan). This preoperative planning software enables the surgeon to simulate the placement of the prosthetic components into their proper positions in the 3D space of the CT data by using a computer-aided design model [17]. Using this software, the postoperative component size and position can also be compared with the position planned preoperatively. Using skeletal reference points, the coordinates of the acetabular and femoral sides can be determined. Each coordinate was also adapted for postoperative implant positioning and alignment evaluation. The cup positioning and alignment were evaluated using a functional pelvic plane coordinate system (Figure 1), and the stem was evaluated using the coordinate system recommended by the International Society of Biomechanics (Figure 2) [22, 23]. Preoperative and postoperative coordinates were unified by an “image matching” system mounted on postoperative evaluation software. The “Image matching” system can automatically superimpose the preoperative and postoperative CT images (Figure 3). The following parameters, that is, implant alignment and positioning were calculated automatically, whereas the component size was calculated manually using postoperative evaluation software [1].

### 2.4. Component Size Accuracy

We investigated the concordance rates of each component size (femoral head, cup, and stem) between 3D CT-based postoperative templating and the actual implant used.

### 2.5. Alignment Measurement

**Figure 4:**
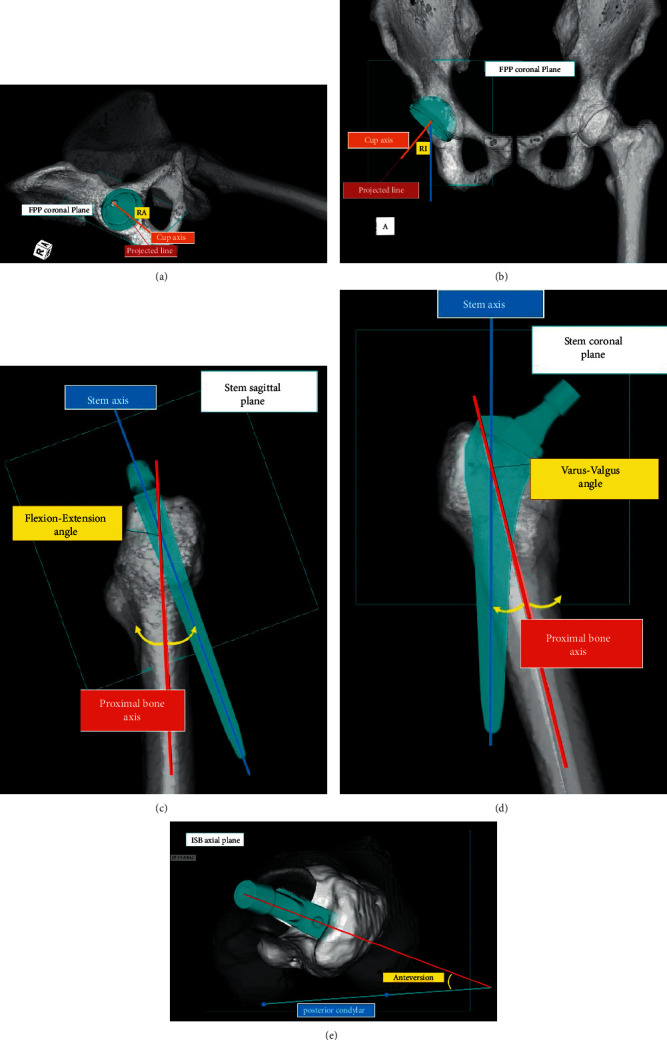
Alignment of components. The radiographic inclination (RI) (a) and radiographic anteversion (RA) (b) of the cup measured according to the acetabular coordinate system. The alignment of the stem measured according to femoral coordinate systems (c), (d), (e).

Radiographic inclination (RI) and radiographic anteversion (RA) were evaluated for the acetabular component alignment. RI is the angle between the acetabular axis and the *Z*-axis projected onto the *XZ* plane, and RA is the angle between the acetabular axis and the *Y*-axis projected onto the *XZ* plane (Figure 4). The definition of three-dimensional acetabular coordinate system was as follows:

 
*X*-axis: the line passing through the left and right ASIS. Positive direction from left to right of the pelvis.
 
*Y*-axis: the line perpendicular to the *X*-axis and the CT coordinate system *Z*-axis and passing through the origin. Positive direction is from the posterior to the anterior of the pelvis.
 
*Z*-axis: A straight line perpendicular to the *X* and *Y* axes. Positive direction is from the inferior to the superior aspect of the pelvis.

Anteversion, varus–valgus angle, and flexion–extension angles were evaluated for the femoral component alignment. Anteversion is the angle between the posterior condylar line and the line from the center of the stem head to the stem axis. The varus–valgus angle is the angle between the proximal bone axis and the femoral component on the coronal plane. The flexion–extension angle is the angle between the proximal bone axis and the femoral component on the sagittal plane (Figure 4). The definition of three-dimensional femoral coordinate systems was as follows.

 
*X*-axis: the line perpendicular to the *Z*-axis and passing through the origin on the plane created by the three points of CFH, ME, and LE. Positive direction is from left to right of the body.
 
*Y*-axis: the line perpendicular to the *Z* and *X* axes and passing through the origin. Positive direction is from the posterior to the anterior of the body.
 
*Z*-axis: the line connecting the center of the femoral head (CFH) and the origin. Positive direction is from distal to proximal femur.

### 2.6. Implant Positioning

For the three-dimensional positioning of the acetabular and femoral components, the distance between the postoperative implant position and the preoperatively planned position was measured. The software used the preoperative plan as a reference point for the coordinates. Three-dimensional distance axes were defined according to acetabular and femoral component coordinate systems: *X*-axis (transverse), *Y*-axis (sagittal), and *Z*-axis (longitudinal) (Figures 1 and 2).

### 2.7. Statistics

The statistical analysis was conducted with JMP® 14 (SAS Institute Inc., Cary, NC, USA). To evaluate the component size accuracy, eight observers performed 3D CT-based postoperative templating without knowing the clinical information. The accuracy was measured with concordance rates of postoperative templating and the actual implant size within a range of ±1 size. The repeatability (intraobserver reliability) and reproducibility (interobserver reliability) of the postoperative evaluation software were calculated using intraclass correlation coefficients (ICC). The measurements were performed by three independent observers (*A*, *B*, and *C*), and two successive measurements were performed at 2-week intervals by one observer (*A*) for the 20 patients. The intraobserver and interobserver differences of alignment measurements and implant position for acetabular and femoral components were calculated. An ICC value of 1 indicated perfect reliability, 0.81–1 was very good, 0.61–0.80 was good, 0.41–0.60 was moderate, and <0.40 indicated poor reliability [24].

## 3. Results

### 3.1. Component Size Accuracy

Table 2 provides the concordance rates of each part. The exact concordance rates of the head, cup, and stem were 96.6% (309/320), 94.7% (303/320), and 97.8% (313/320), respectively. The concordance rates of postoperative templating within a range of ±1 size was 100% in all components.

### 3.2. Alignment Measurement

Table 3 provides the intraobserver and interobserver ICC for alignment measurement. The intraobserver and interobserver ICC were 0.964 and 0.969 in RA, respectively, and 0.879 and 0.827 in RI, respectively, for the acetabular component. The intraobserver and interobserver ICC were 0.996 and 0.995, respectively, in anteversion; 0.973 and 0.970, respectively, in varus–valgus angle; and 0.993 and 0.989, respectively, in flexion–extension angle for the femoral component.

### 3.3. Implant Positioning

Table 4 provides the interobserver and intraobserver ICC for implant positioning. For the acetabular component, the intraobserver and interobserver ICC were, respectively, 0.987 and 0.987 in the *X*-axis (transverse), 0.988 and 0.987 in the *Y*-axis (sagittal), and 0.961 and 0.958 in the *Z*-axis (longitudinal). For the femoral component, the intraobserver and interobserver ICC were, respectively, 0.879 and 0.841 in the *X*-axis (transverse), 0.993 and 0.956 in the *Y*-axis (sagittal), and 0.995 and 0.965 in the *Z*-axis (longitudinal).

## 4. Discussion

The present study investigated the component size accuracy and the intraobserver and interobserver reliabilities of CT-based postoperative evaluation software in THA. The results showed that postoperative evaluation software can evaluate postoperative implant size and orientation with perfect to very good reliability.

The use of two-dimensional plain radiography has been the standard for the evaluation of the acetabular component. RI is measured between the long axis of the implant and the tear-drop line. The ratio of the short and long axes of the implant is widely used for assessing the anteversion [11]. Some reports have suggested that the reliability of the acetabular component measurement on plain radiographs using a normal PACS system is high [25–27]. The EOS system is a novel imaging method that uses biplanar low-dose X-rays, which can evaluate implant alignment semiautomatically [15]. Lazennec et al. reported that the reliability of conventional acetabular component measurement on plain radiographs was lower than the performance of the EOS system [12]. However, the measurement of RA is an issue that is yet to be addressed. In the present study, we demonstrated very good reproducibility for RA. Since the pelvic coordinate axis is set after CT imaging and is available as a reference, it is not affected by posture and limb position. We found a slight decrease in the reliability of inclination. With this system, the reference point for the distal implant edge can affect the inclination and positioning on the *Z*-axis. The relatively lower reliability of the inclination and *Z*-axis indicates that the reference point may vary.

Although several reports on the reliability of evaluation methods after THA have concerned acetabular components, there are a few reports on the femoral component [15, 16, 26]. Some studies focused on the stability of the component and the bone reaction of the femur [28, 29]; however, there have been no studies that explore the reliability of the femoral component varus–valgus and flexion–extension angles. The alignment of the femoral component is, however, assumed to be important in preventing impingement and dislocation of the femoral head [30]. Lee et al. reported that the reliability of identifying stem anteversion by plain radiographs is high; intraobserver reliability was 0.944, and interobserver reliability was 0.934 [26]. Another study that used the EOS system reported an intraobserver reliability of 0.998 and an interobserver reliability of 0.997. The reliability of femoral component anteversion in the present study is equal to or better than other methods. Additionally, with this method, the proximal bone axis and 3D coordinate system of the femur can be placed automatically from the CT image, so it is not affected by the orientation of the lower limbs.

The 3D position of each implant is assumed to affect the biomechanics of the joint [31], the impingement of components, and leg length [32]. Therefore, the 3D position of each implant is also measured and installed via navigation [33]. Nevertheless, there are few reports on its positional evaluation method [34]. Leg length is measured from the transverse line, and femoral offset is measured from the hip center on the X-ray. However, the sagittal positional shift (*Y*-axis, in this study) cannot be evaluated without a 3D approach. This method, therefore, can be useful in evaluating the leg length discrepancy or postoperative biomechanics of the hip joint.

This system allows us to simulate the range of motion and evaluate impingement after surgery. Precise evaluation of the implant position and alignment with residual osteophytes after surgery, identified by postoperative CT, can enable the assessment of the risk of dislocation due to impingement. It can also provide information that is useful for patient guidance. Another potential clinical advantage of this system is the long-term evaluation of implant stability because this method can evaluate implant alignment and position three-dimensionally; it would be possible to detect even slight loosening. In the case of revision THA, knowledge of the diameter of the existing cup can be very important. The knowledge of the correct size of the acetabular cup can decrease intraoperative complications by having the correct implant removal equipment size selected.

Our method has several limitations. First, our evaluation involved only one type of implant, and we do not know what the results of the evaluation with other implants would show. Most acetabular components are spherical; therefore, our results might be generalizable to other acetabular components. Second, the implant size was blinded. Knowing the size, however, might improve reliability because we could calibrate for the implant size making it easier to determine the location of the implant edge. However, we believe that, compared with most studies, the present study has been performed under stricter conditions.

## 5. Conclusion

The CT-based postoperative evaluation software was able to evaluate the position of the implant with high reproducibility. It can, therefore, be a useful tool for evaluating the accuracy of implant size and placement after primary THA and before revision THA.

## Figures and Tables

**Table 1:** Demographics of all patients for the postoperative analysis.

Characteristic (*n* = 20)	Mean (SD)
Age at CT (years)	64.8 (11.5)
Sex (male), *n*	8
Weight (kg)	59.2 (12.6)
Height (*m*)	1.53 (11.5)
Body mass index (kg/m^2^)	25.1 (4.2)
Diagnosis	Number (rate)
Osteoarthritis	15 (75%)
Osteonecrosis	5 (25%)
Shape of the femoral canal	Number (rate)
Champagne flute (CFI > 4.7)	3 (15%)
Normal (3.0 ≦ CFI ≦ 4.7)	14 (70%)
Stovepipe (CFI < 3.0)	3 (15%)

CFI, canal flare index.

**Table 2:** Component size accuracy of templating.

	−1 size	0 size	+1 size
Head	2 (0.6%)	309 (96.6)	9 (2.8)
Cup	0 (0%)	303 (94.7%)	17 (5.3%)
Stem	5 (1.6%)	313 (97.8%)	2 (0.6%)

**Table 3:** Interobserver and intraobserver reliabilities for alignment measurement.

		Average (SD)	Intraobserver reliability (CI)	Interobserver reliability (CI)		
				A1-B	A1-C	B-C
Cup	RA	17.6 (4.3)	0.972 (0.933–0.989)	0.978 (0.943–0.991)	0.970 (0.915–0.989)	0.982 (0.955–0.993)
	RI	36.6 (3.8)	0.955 (0.893–0.982)	0.898 (0.765–0.958)	0.965 (0.913–0.986)	0.892 (0.753–0.956)

Stem	Anteversion	27.6 (13.1)	0.993 (0.984–0.997)	0.999 (0.998–0.999)	0.999 (0.997–1.000)	0.999(0.997–0.999)
	Varus–valgus angle	−1.2 (1.7)	0.956 (0.894–0.982)	0.987 (0.877–0.995)	0.991 (0.977–0.996)	0.995 (0.988–0.998)
	Flexion–extension angle	1.8 (2.3)	0.991 (0.980–0.997)	0.994 (0.985–0.998)	0.995 (0.986–0.998)	0.997 (0.992–0.999)

SD, standard deviation; CI, 95% confidence interval; RA, radiographic anteversion; RI, radiographic inclination.

**Table 4:** Intraobserver and interobserver reliabilities for implant positioning.

		Average (SD)	Intraobserver reliability (CI)	Interobserver reliability (CI)		
				A1-B	A1-C	B-C
Cup	*x* (transverse)	0.8 (2.8)	0.992 (0.981–0.997)	0.987 (0.965–0.998)	0.989 (0.974–0.996)	0.948 (0.875–0.979)
	*y* (sagittal)	0.2 (2.5)	0.992 (0.979–0.997)	0.987 (0.968–0.995)	0.982 (0.952–0.993)	0.853 (0.665–0.939)
	*z* (longitudinal)	−1.2 (2.0)	0.966 (0.917–0.986)	0.947 (0.865–0.979)	0.960 (0.900–0.984)	0.834 (0.626–0.931)

Stem	*x* (transverse)	−0.9 (2.0)	0.976 (0.943–0.991)	0.977 (0.938–0.991)	0.978 (0.946–0.991)	0.987 (0.965–0.995)
	*y* (sagittal)	1.0 (2.8)	0.993 (0.983–0.997)	0.993 (0.983–0.997)	0.992 (0.981–0.997)	0.995 (0.988–0.998)
	*z* (longitudinal)	0.2 (5.1)	0.996 (0.990–0.998)	0.996 (0.990–0.998)	0.996 (0.990–0.998)	0.997 (0.992–0.999)

SD, standard deviation; CI, 95% confidence interval.

## Data Availability

The data used to support the findings of this study are available from the corresponding author upon request.
